# Supplementary material for: Moulding three-dimensional curved structures by selective heating
Source: R Soc Open Sci. 2020 Feb 26;7(2):200011. doi: 10.1098/rsos.200011 (PMC7062067; doi:10.1098/rsos.200011)
Supplement: Supplementary Information: Molding 3D curved structures by selective heating [file rsos200011supp1.pdf]

Supplementary Information: Molding 3D curved structures by selective heating

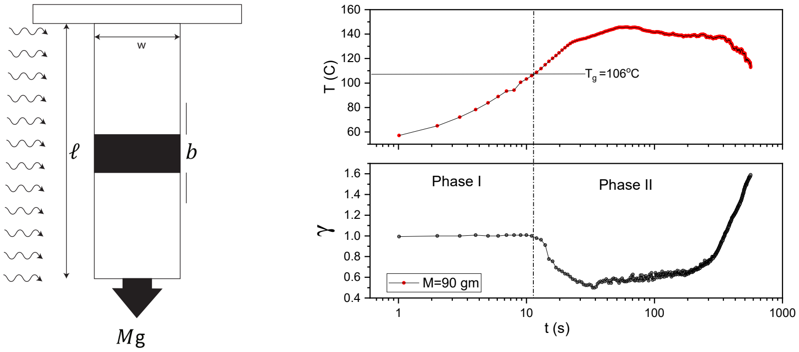

Figure 1: **Material Properties 1**: Shrinky Dinks are pre-stretched polystyrene sheets which are cross linked and stretched above their glass transition temperature and then cooled rapidly to be frozen in the stretched state. The material becomes soft and rubbery above the glass transition temperature. We did the following experiment to measure the thermo-mechanical properties of the material. We took a rectangular piece of the material (shrinky dink) of dimension 30 mm in width, 130 mm in length. A black patch of 30 mm by 30 mm was drawn across the width of this piece. This rectangular piece was hung vertically and a load M was attached to it. The system was heated by an infrared lamp. The deformation and the temperature of the strip was monitored simultaneously by a visible range (NIKON D750 DSLR) and an Infrared camera (FLIR- A600), respectively. The experimental setup is shown schematically in the left panel. With time t, the temperature T of the black portion increases. In the initial phase when $t < 10\,\text{s}$ and $T < 106°\text{C}$ almost no visible deformation happens. Beyond $t = 10\,\text{s}$, the temperature T is observed to be greater than $106°\text{C}$. In this phase, (i) the material becomes soft and rubbery (ii) the release of the recovery stress from the cross linking pulls the weight upwards and (iii) the temperature-induced deformation reaches a plateau. The width of this phase in time depends on the load, e.g., for a $90\,\text{gm}$ load this is about $100\,\text{s}$ while for a $250\,\text{gm}$ load it is about $10\,\text{s}$ (data not shown). In the long run, the material begins to flow. In the right panel we plot the variation of the contraction coefficient γ and the temperature T of the black region.

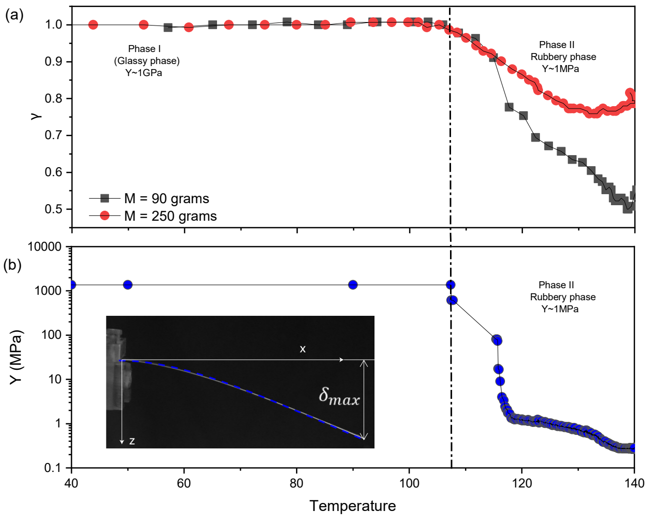

Figure 2: **Material Properties 2:** In (a) and (b) we plot the variations of γ and the elastic constant Y, as functions of temperature. Below 106°C the material is glassy and its elastic constant is of the order of 1 GPa and above 106°C the material becomes rubbery and the value of Y drops to about 1 MPa. We use the method of deflection of a cantilever to find out the Youngs Modulus of the Shrinky Dink material in Phase I. By this method $Y = \frac{3Mg\ell^3}{2\delta_{max}wh^3} \simeq 1\,\text{GPa}$. Here δ_{max} is the maximum deflection of the cantilever, $\frac{Mg}{\ell}$ is the load per unit length, $\ell = 130\,\text{mm}$ is the length, $w = 30\,\text{mm}$ is the width and $h = 0.26\,\text{mm}$ is the thickness of the cantilever. The image in the inset of (b) shows the bending of a strip of the Shrinky Dink due to its own weight. The dashed line shows the expected functional form $z(x) = \frac{mgx^2}{2Ybh^3}(x^2 + 6\ell^2 - 4x\ell)$, for the deflection $z(x)$ of the the cantilever as a function of its distance x from the clamped end. Here m is mass per unit length of the cantilever. In the rubbery phase, the change in the length is brought about by the combined effect of the recovery force exerted by the material and the tensile force exerted by the load. The variation of the elastic modulus Y with temperature was evaluated by obtaining the temperature dependent contraction coefficients $\gamma_1(T)$ and $\gamma_2(T)$ for two different applied loads M_1g and M_2g at different temperatures from the following equation $Y = \left(\frac{\gamma_1\gamma_2(M_1-M_2)}{\gamma_1-\gamma_2}\right)\left(\frac{g}{wh}\right)$. Here $M_1 = 250\,\text{gm}$ and $M_2 = 90\,\text{gm}$.

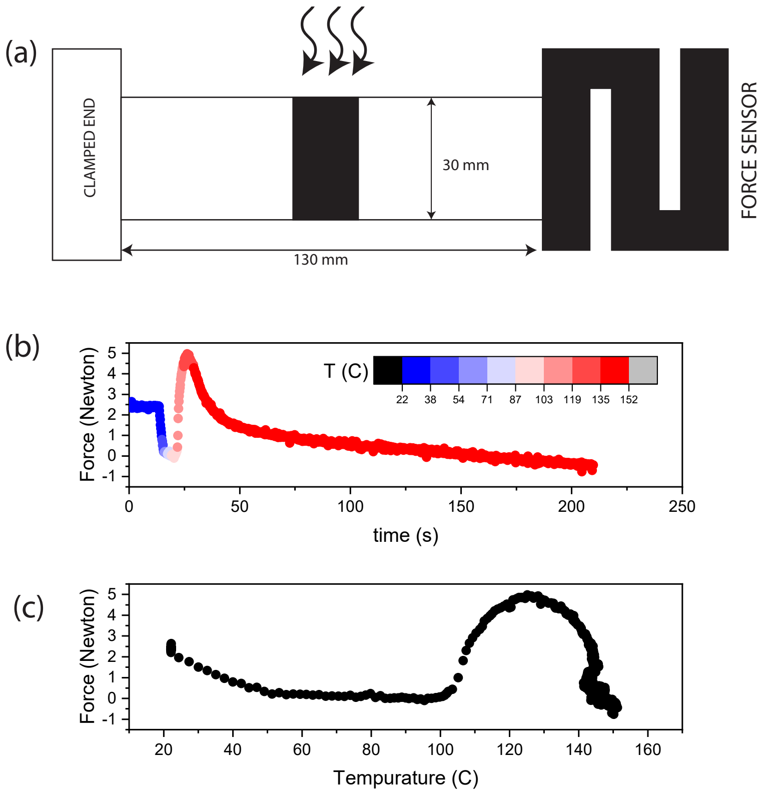

Figure 3: **Material Properties 3:**(a) The figure schematically shows the experimental setup to measure the recovery force generated by the material when heated. A rectangular strip with a black patch similar to that described in Fig 1 was held taut between two clamped ends. One of the clamped ends was attached to a 5 Kg load cell. The black portion of the strip was heated selectively by an IR lamp. The temperature of the black portion was monitored by the IR camera. The figure (b) shows the variation of the force measured by the sensor as a function of time. The color of the symbol represents the temperature of the black portion. The initial drop is related to the loss of tension (tautness) in the material. The figure (c) shows the variation of the force with temperature. Around the glass transition temperature of 106°C the recovery force builds up. In the long run, as the temperature exceeds 130°C, the material begins to flow and the recovery force decreases. The peak recovery stress is about $F/wh = 0.5\,\mathrm{MPa}$ where $w = 30\,\mathrm{mm}$ and $h = 0.26\,\mathrm{mm}$.

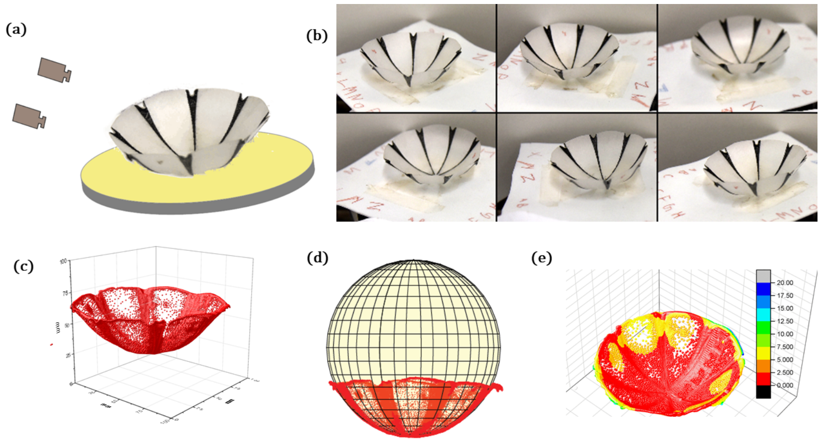

Figure 4: **The degree of error in the molded structure :** To quantify the error in the molded structure we first obtained its 3-Dimensional coordinate description. This was done by taking multiple photographs from two viewpoints. We use a NIKON D750 DSLR camera to do this. A diffused light source was used for illumination. A schematic description of the experimental setup for taking these images is shown in (a). We placed the molded object on a rotating platform. For each viewpoint, a series of images were takes for every 10 degree movement of the rotating platform. The images in (b) are that of a part of a sphere molded by the tailoring method, taken from different viewpoints. Many such images were then combined to form a set S of points (x_i, y_i, z_i) that lie on the moulded shape using the Autodesk Recap software. (c) The figure shows the set S as a 3D scatter plot. The set S was translated and rotated so as to obtain the best overlay with the image of the target function $\varphi : D \to M$. The panel (d) shows an example of such overlay. The percentage error $\left|\frac{z_i - \varphi(x_i, y_i)}{d}\right| \times 100$, where d is the diameter of the object (maximum possible distance between pairs of points), is colour-coded on each point of S. For the case of the sphere this error is shown in (e).

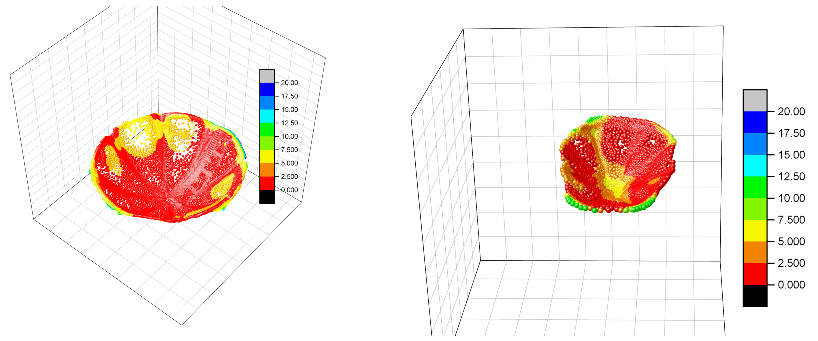

Figure 5: **The error in the molded structure using contraction-tailoring:** The left and the right panels show the percentage error in the height of the shapes molded using contraction-tailoring method. The left panel quantifies the error in molding a portion of a sphere and the right panel quantifies the error in molding a portion of an ellipsoid. These correspond to the shapes shown in Fig 5(a) and Fig 7(b) of the main manuscript. The method of calculating the error is described in the caption of Fig.4 of the supplementary material.

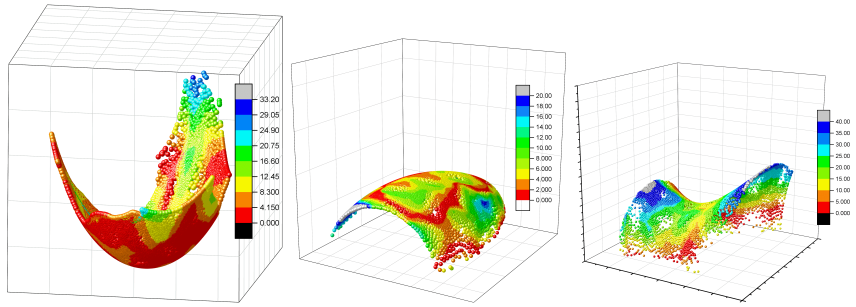

Figure 6: **The error in the distance molded structure :** The panels shows the percentage error in the height of the shapes molded using the distance molding method. The left, center and the right panels quantify the error in molding a portion of a sphere, a portion of a spindle and a portion of a saddle. These corresponds to the shapes shown in Fig 11 of the main manuscript. The method of calculating the error is described in the caption of Fig.4 of the supplementary material.

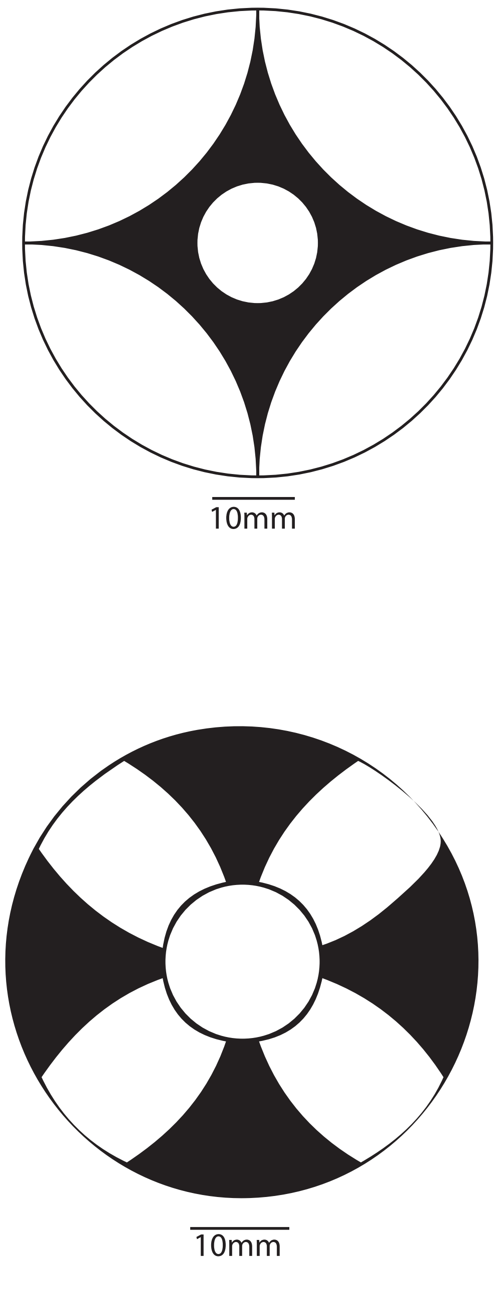

Figure 7: Input pattern of Fig 2b of the main manuscript.

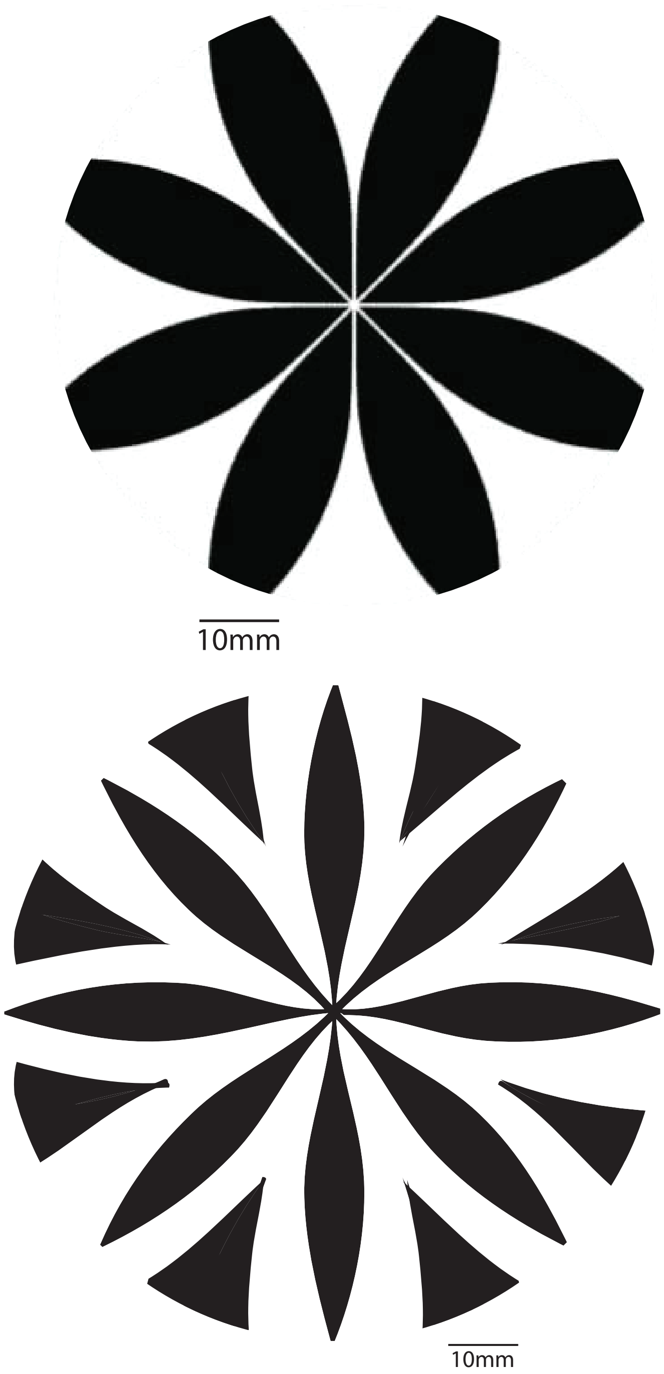

Figure 8: Input pattern of Fig 5a (top panel) and Fig 5(b) (bottom panel) of the main manuscript.

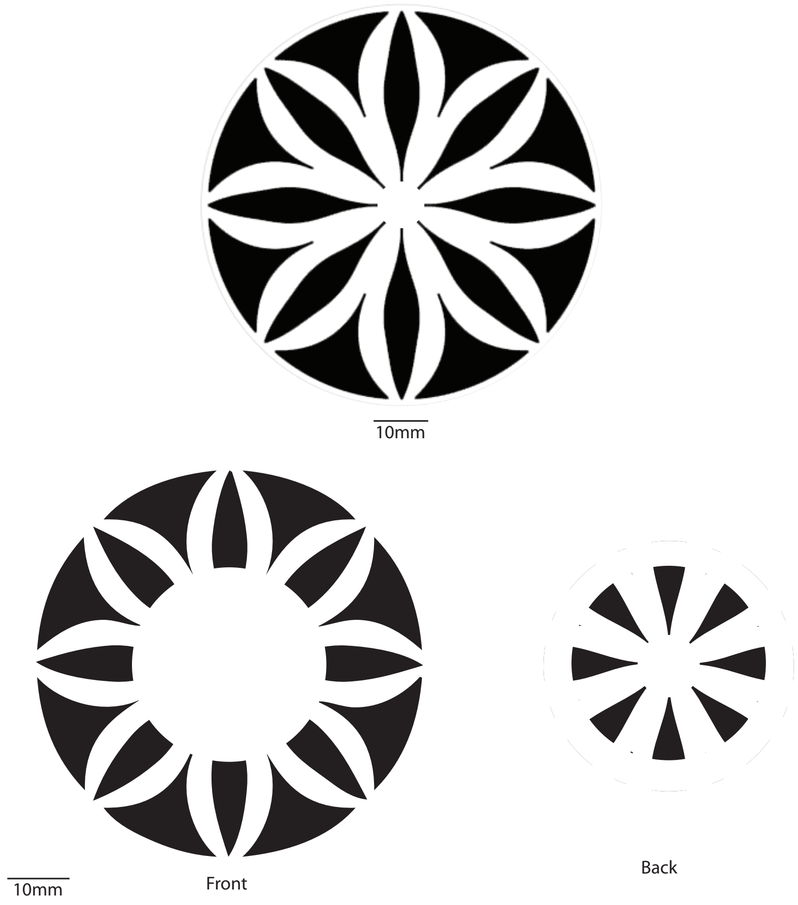

Figure 9: Top panel: This pattern produces the shape shown in Fig 6 (c) in the main manuscript. Bottom panel: The patterns marked ‘back’ and ‘front’ is printed on opposite side of the plastic. The two images are arranged in an concentric manner. On heating combination of these two patterns produce the shape shown in Fig 6 (d) in the main manuscript.

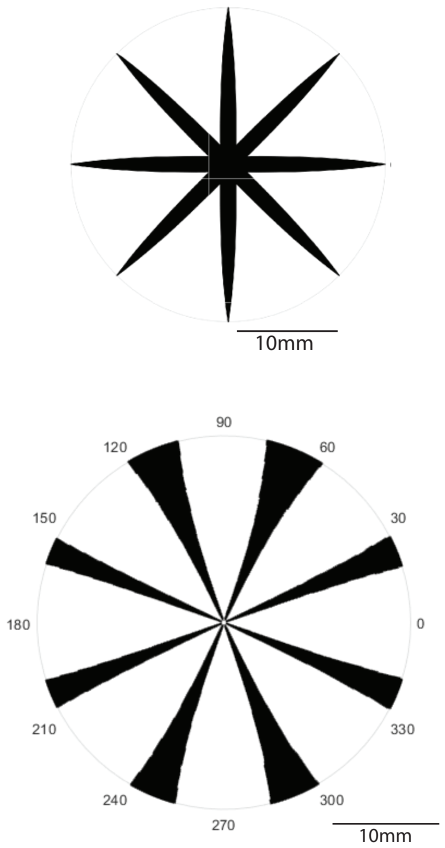

Figure 10: The top pattern forms the input of Fig 7(a) of the main manuscript. The bottom pattern forms the input of Fig 7(b) of the main manuscript

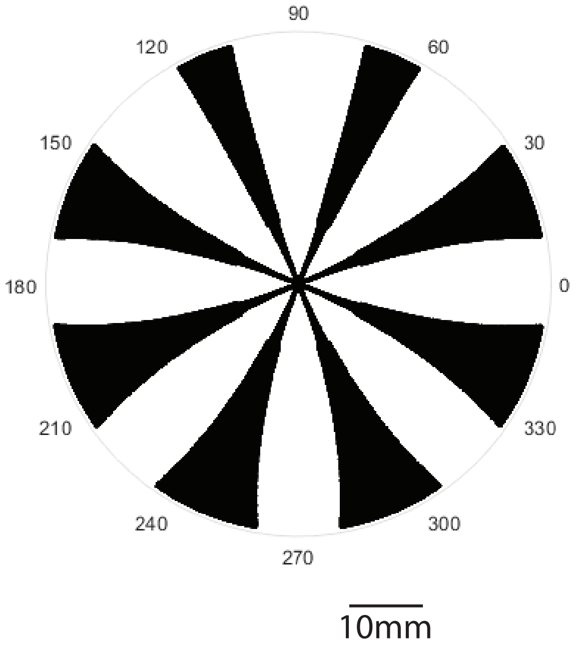

Figure 11: The pattern forms the input of the Fig 7(c) of the main manuscript.

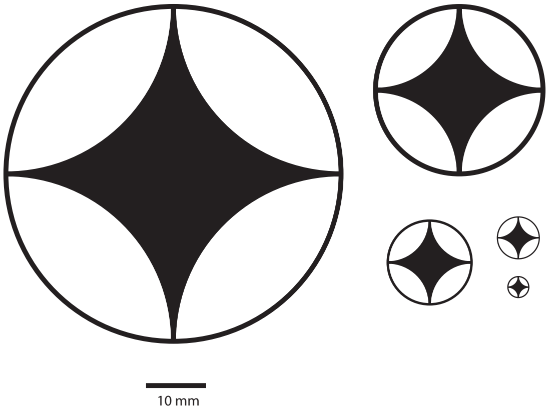

Figure 12: The pattern forms the input of the Fig 7(d) of the main manuscript.

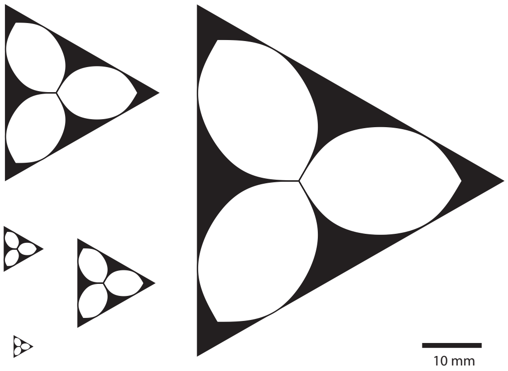

Figure 13: The pattern forms the input of the Fig 7(e) of the main manuscript.

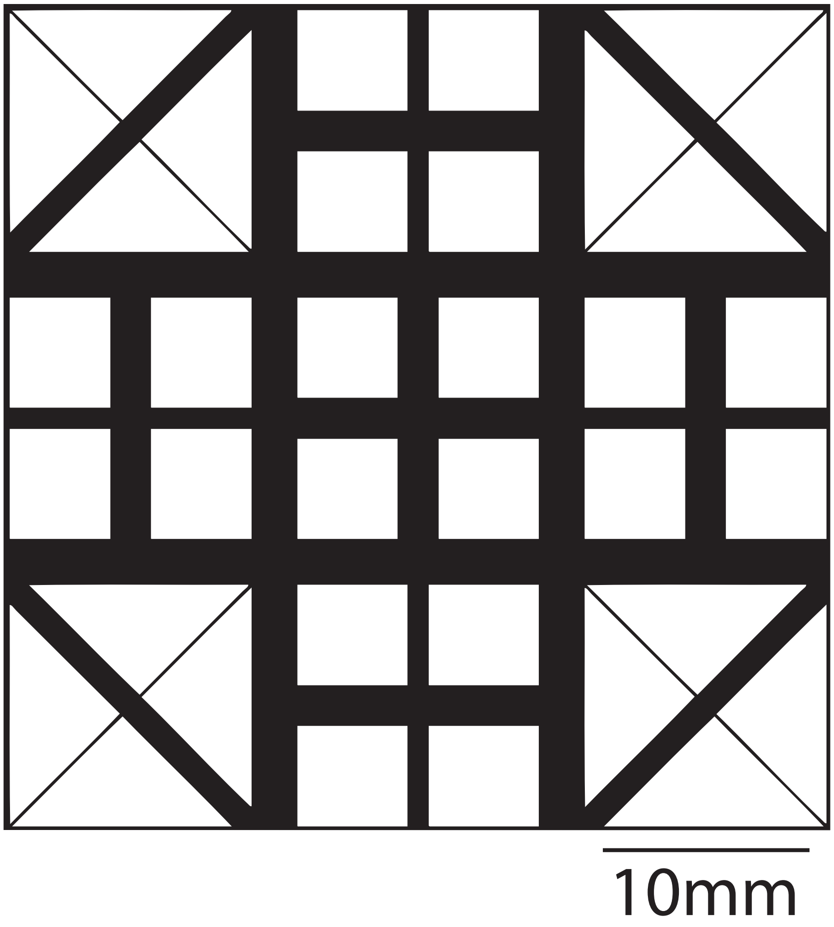

Figure 14: The pattern forms the input of the Fig 9(a) of the main manuscript.

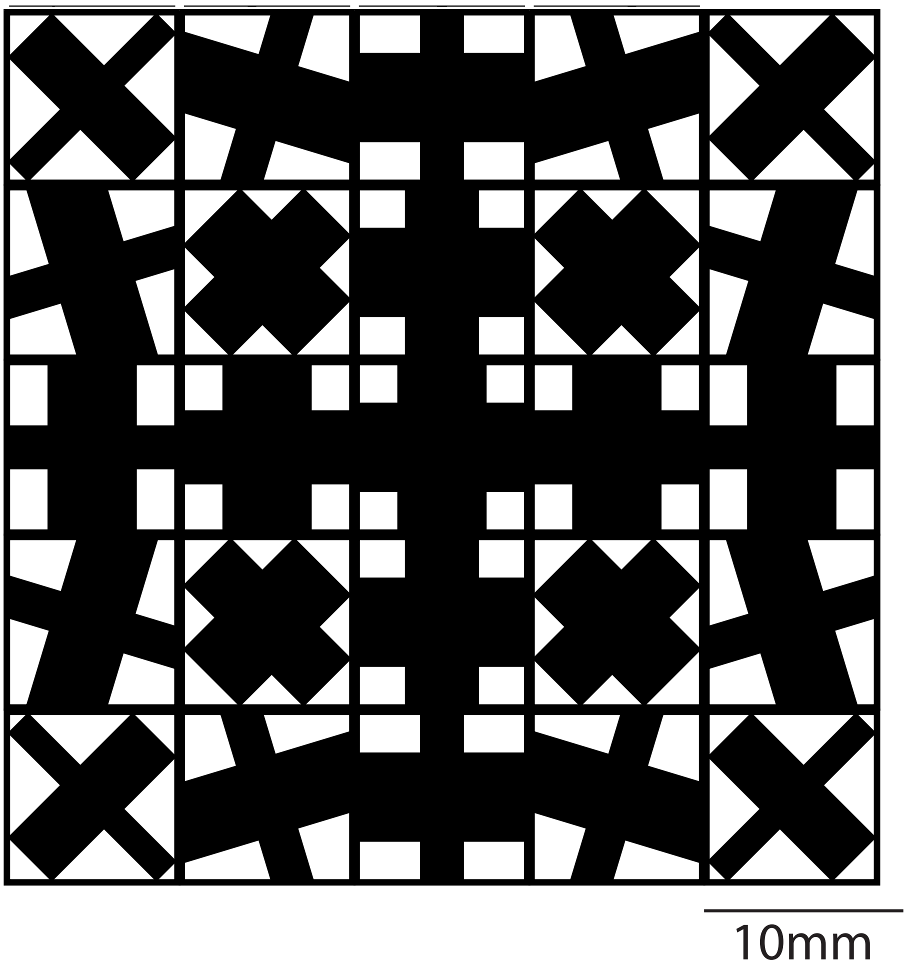

Figure 15: The pattern forms the input of the Fig 9(b) of the main manuscript.

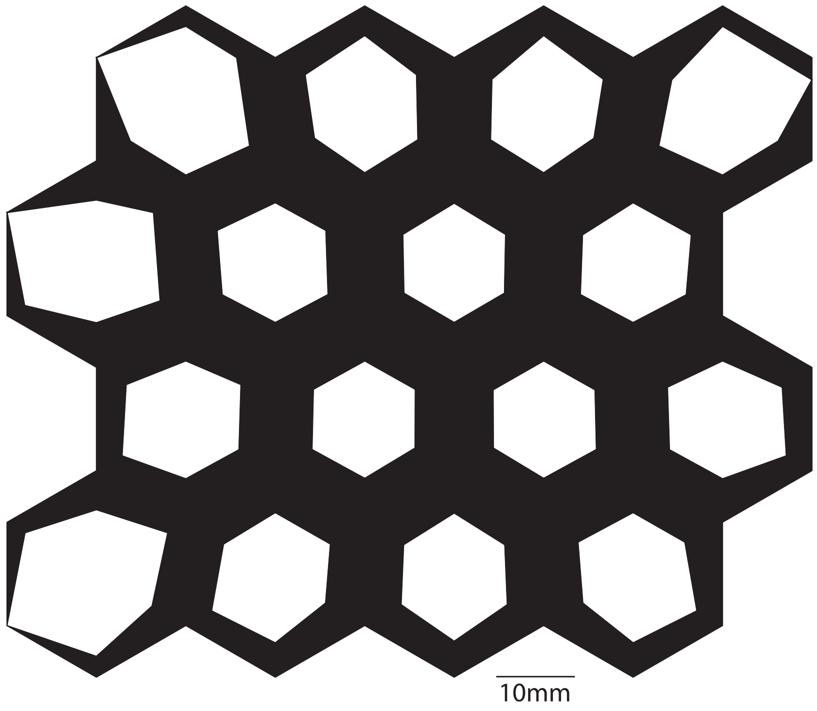

Figure 16: The pattern forms the input of the Fig 11(a) of the main manuscript.

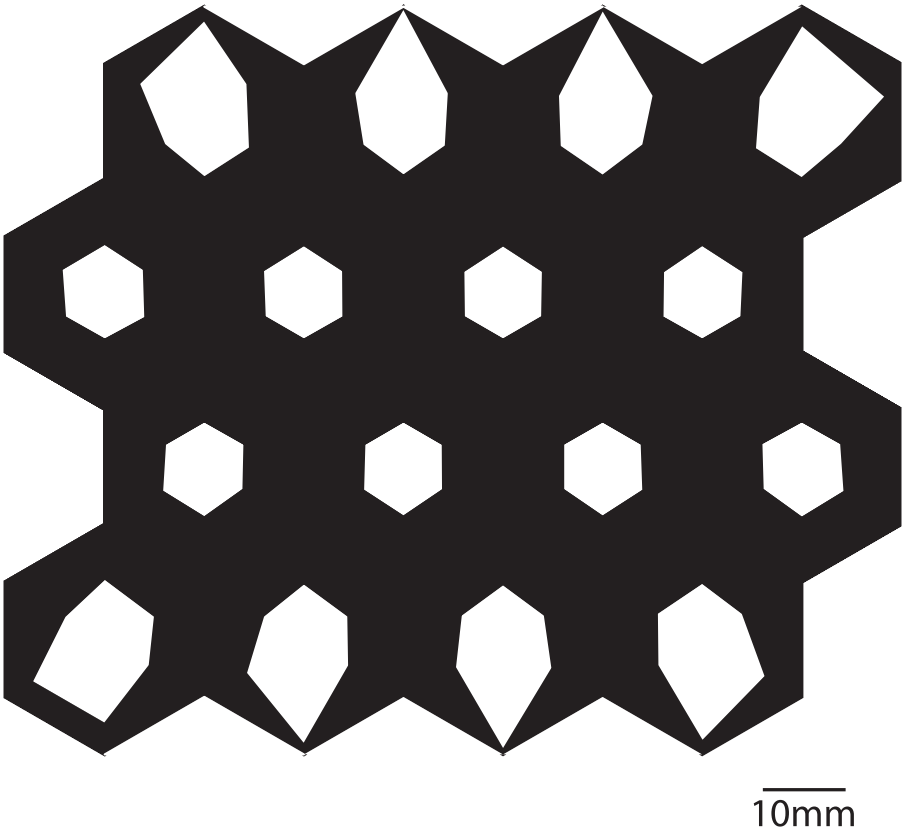

Figure 17: The pattern forms the input of the Fig 11(b) of the main manuscript.

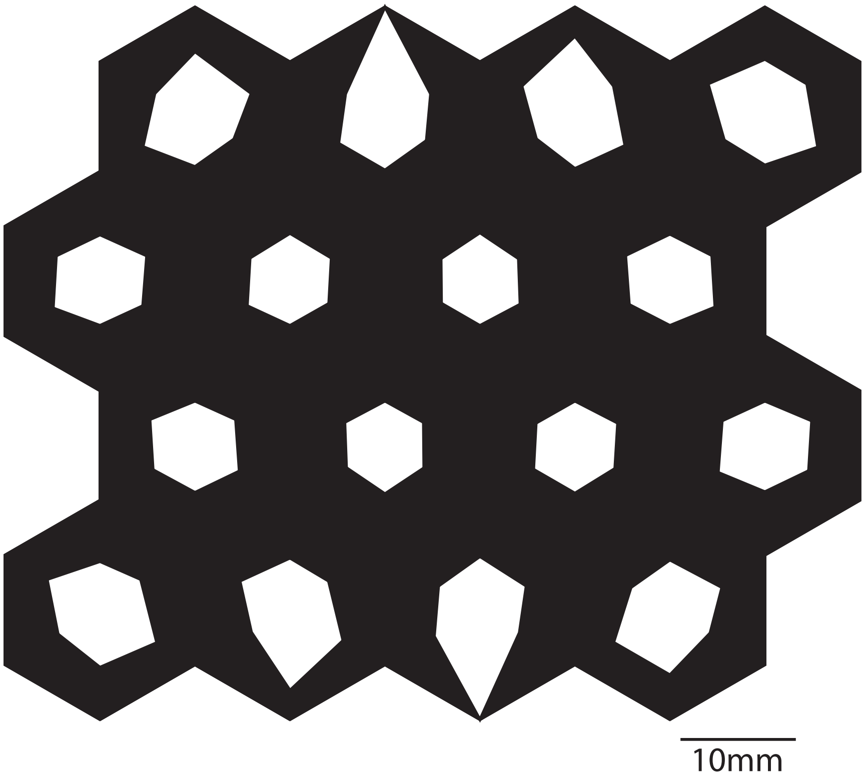

Figure 18: The pattern forms the input of the Fig 11(c) of the main manuscript.
